# Promoting rational antibiotic therapy among high antibiotic prescribers in German primary care—study protocol of the ElektRA 4-arm cluster-randomized controlled trial

**DOI:** 10.1186/s13012-022-01241-4

**Published:** 2022-10-04

**Authors:** Christin Löffler, Theresa Buuck, Julia Iwen, Maike Schulz, Antonia Zapf, Peter Kropp, Anja Wollny, Linda Krause, Britta Müller, Ann-Katrin Ozga, Elisabeth Goldschmidt, Attila Altiner

**Affiliations:** 1 Institute of General Practice, Rostock University Medical Center, Postbox 100888, 18055 Rostock, Germany; 2 Association of Substitute Health Funds (Vdek) e.V, Berlin, Germany; 3 Central Research Institute of Ambulatory Health Care in Germany (Zi), Berlin, Germany; 4 Institute of Medical Biometry and Epidemiology, University Medical Center Hamburg-Eppendorf, Hamburg, Germany; 5 Institute of Medical Psychology and Medical Sociology, Rostock University Medical Center, Rostock, Germany; 6 Society for Patient-Centred Communication (GPZK e.V.), Rostock, Germany

**Keywords:** Antibacterial agents, Respiratory tract infection, Urinary tract infections, Continuing medical education, Primary care, Physician-patient relation, Shared decision-making, Antibiotic resistance

## Abstract

**Background:**

The rational use of antibiotics is of great importance in health care. In primary care, acute respiratory infections are the most common cause of inappropriate antibiotic prescribing. Since existing studies aiming to optimize antibiotic use are usually based on the voluntary participation of physicians, general practitioners (GPs) with inappropriate prescribing behavior are underrepresented. For the first time in Germany, the ElektRA study will assess and compare the effects of three interventions on antibiotic prescribing rates for respiratory and urinary tract infections among high prescribers in primary care.

**Method:**

ElektRA is a 4-arm cluster-randomized controlled trial among German GPs in nine regional Associations of Statutory Health Insurance Physicians. On their behalf, the Central Research Institute of Ambulatory Health Care in Germany (Zi) analyses all outpatient claims and prescription data. Based on this database, high antibiotic prescribing GPs are identified and randomized into four groups: a control group (*N*=2000) and three intervention arms. We test social norm feedback on antibiotic prescribing (*N*=2000), social norm feedback plus online training on rational prescribing practice and communication strategies (*N*=2000), and social norm feedback plus online peer-moderated training on rational antibiotic prescribing, communication strategies, and sustainable behavior change (*N*=1250). The primary outcome is the overall rate of antibiotic prescriptions. Outcomes are measured before intervention (T0, October 2020–September 2022) and over a period of 15 months (T1, October 2022 to December 2023) after randomization.

**Discussion:**

The aim of the study is to implement individualized, low-threshold interventions to reduce antibiotic prescribing among high prescribers in primary care. If successful, a change in behavior among otherwise difficult-to-reach high prescribers will directly improve patient care. The increase in quality of care will ideally be achieved both in terms of the quantity of antibiotics used as well as the kind of substances prescribed. Also, if effective strategies for high prescribers are identified through this study, they can be applied not only to the antibiotics addressed in this study, but also to other areas of prescription management.

**Trial registration:**

Current Controlled Trials ISRCTN95468513.

**Supplementary Information:**

The online version contains supplementary material available at 10.1186/s13012-022-01241-4.

Contributions to the literature

- Previous studies on reducing antibiotic prescribing in primary care are based on voluntary participation. Physicians with high prescribing behavior are underrepresented.
- ElektRA specifically targets “high prescribers” who often lack sensitivity to the issue and perceive their own prescribing behavior as unproblematic.
- To our knowledge, no study has yet attempted to identify and optimize high antibiotic prescriptions in primary care in Germany.
- We test usual care in comparison to three interventions that include social norm feedback on antibiotic prescribing, communication strategies, and sustainable behavior change through letters, online training, and peer-moderated online training.

## Background

Antibiotics are an essential part of therapy for severe bacterial infections. However, their effectiveness is threatened by increasing levels of antibiotic resistance [1]. Therefore, before prescribing antibiotics, the benefits of antibiotic use should be weighed against possible harm, both from a micro and a macro perspective. Inappropriate antibiotic use as well as the non-rational prescription of broad-spectrum and/or reserve substances should be avoided.

In Germany—as in many other Western countries—80–90% of antibiotics are prescribed in primary care [2]. Thereby, acute respiratory tract infections (ARTI) are the most frequent cause of the prescription of antibiotics. However, in many of these cases, antibiotic use is unnecessary or inappropriate. While most physicians have a moderate prescription rate, there is a group of physicians who, compared to their colleagues, disproportionately prescribe antibiotics in general or certain groups of active agents. In 2015, almost one third of German general practitioners (GPs) belonged to the group of so-called high prescribers [2]. In the last years, various studies and initiatives have addressed the issue of rational antibiotic use and attempted to influence prescription rates at the regional and national levels through awareness-raising, knowledge transfer, and training [3–6]. However, all these studies were based on voluntary participation. Consequently, high prescribing physicians are underrepresented, and the studies are therefore prone to selection bias. According to current knowledge, the main reasons for the lack of participation of physicians with high prescription rates of antibiotics in studies are their lack of sensitivity to the topic and the perception that their own prescribing behavior is unproblematic [2]. International evidence shows that high prescribers can be motivated to change their prescribing behavior through targeted approaches [7–10]. However, the measures used differ in their approach and effectiveness and are partly dependent on the functioning and organization of the respective health system. To our knowledge, no study has sought to identify and optimize high antibiotic prescribing in primary care in Germany.

### Objectives

The aim of the ElektRA (“Elektive Förderung Rationaler Antibiotikatherapie”—Elective Promotion of Rational Antibiotic Therapy) study is to identify which of the interventions tested in other countries is transferable to the German health care setting and best at gaining access to the group of high prescribers of antibiotics and at sustainably reducing the antibiotic prescription rate for typical treatment occasions such as acute infections. Because of their importance in primary care, we focus on respiratory and urinary tract infections [11]. In addition, the aim is to promote the quality of prescribing with regard to the choice of an active agent. The primary outcome of the study is the overall rate of antibiotic prescriptions, measured as the proportion of patients with antibiotic prescriptions out of all patients with a prescription. Secondary outcomes are the overall prescription rates before and after the intervention for cephalosporins and fluoroquinolones separately. Also, to be explored is whether there are differences in the level of the effects achieved among groups. Factors explored will include region, patients’ multimorbidity, indications of the respective active agent, and failure of previous therapy. A mixed methods evaluation is also carried out.

## Methods

### Trial design

The ElektRA study is a 4-arm cluster-randomized controlled trial addressing high antibiotic prescribers among German GPs of nine regional Associations of Statutory Health Insurance Physicians (ASHIP). On their behalf, the Central Research Institute of Ambulatory Health Care in Germany (Zi) analyses all outpatient claims and prescription data (T0, October 2020–September 2022). Based on this data set, we can identify GPs who use antibiotics or problematic agents (e.g., fluoroquinolones) more frequently than their colleagues. From this pool, GPs are randomized into four groups: a non-contacted control group and three intervention arms (A–C). Within these arms, we test (A) *social norm feedback* on antibiotic prescribing, (B) *social norm feedback* plus *online training* on rational prescribing practice and communication strategies, and (C) *social norm feedback* plus *online peer-moderated training* on rational antibiotic prescribing, communication strategies, and sustainable behavior change. Randomization is stratified by region. GPs in groups A–C will be contacted by their respective regional ASHIP. The primary outcome is the overall rate of antibiotic prescriptions, measured as the proportion of patients with antibiotic prescriptions out of all patients with a prescription. Outcomes are measured over a period of 15 months (T1, October 2022–December 2023) after randomization. In addition, a mixed-method process evaluation is carried out.

### Data basis

The data basis is the pseudonymized German prescription data (according to § 300 SGB V) and outpatient claims data (according to § 295 SGB V). Since the processing of these data takes place within the framework of tasks of the regional ASHIP defined in the German Social Security Code SGB V, processing of the data is possible without a declaration of consent. Despite the fact that an ethics vote is not mandatory for secondary data analyses [12], it was obtained for the evaluation study in general from the Ethics Committee of the Rostock University Medical Center. Since no informed consent must be obtained before randomization, ElektRA has a real control group that is not aware of the study and the interventions. Distortions due to the Hawthorne effect are thus excluded. A selection bias can also be ruled out among the intervention participants, since not only GPs who are motivated and interested receive an intervention. For the evaluation, a total of at least 13 quarters are analyzed. To ensure that the prescribing behavior before the intervention can be examined sufficiently and independently of seasonal fluctuations, at least 8 quarters are used to determine the baseline. For the follow-up data from 5 quarters are available.

### Participants

The Zi uses a summative score to determine potentially eligible GPs and GP practices based on German prescription and outpatient claims data before intervention (T0, October 2020–September 2022). The maximum score value is six. A GP or GP practice obtains one additional point if its own value in one of the aspects exceeds the 75% percentile of antibiotic prescribing within the respective region. Here, a region represents the area for which a participating ASHIP is responsible. In Germany, there are in total of 17 of those regions, 9 of which are participating in this study. For inclusion, more than two points are necessary. The aspects listed in Table 1 are used for score formation.

**Table 1:** Aspects considered for score formation

1	Overall prescription rate (ratio of patients with antibiotic prescriptions to patients with any prescription)
2	Prescription rate for upper respiratory tract infections (ratio of patients with upper respiratory tract infection to antibiotic prescriptions for these patients)
3	Prescription rate for lower respiratory tract infections (ratio of patients with lower respiratory tract infection to antibiotic prescriptions for these patients)
4	Prescription rate of fluoroquinolones (2 points possible): total prescription rate of fluoroquinolones (ratio of patients with fluoroquinolone to patients with antibiotic) and/or fluoroquinolone prescription rate for urinary tract infection (ratio of urinary tract infection patients with fluoroquinolone to urinary tract infection patients with antibiotics)
5	Prescription rate of cephalosporins (ratio of patients with cephalosporin to patients with antibiotics)

After identification, the GPs or GP practices meeting the inclusion criteria are randomized and stratified by region. As identification is based on pseudonymized data, an independent trust center will generate study pseudonyms that will enable all participating regional ASHIP and the Zi to communicate about identified entities and allow the ASHIP to re-identify these eligible GPs or GP practices and contact them. The Zi on the other hand will always exclusively work with pseudonymized data to compile all further analyses, e.g., those necessary for providing GP-specific prescription feedback. Physicians who have already participated in the RESIST Innovation Fund project [13] are excluded because they have already received e-learning on optimizing antibiotic prescribing.

### Recruitment

Nine regional ASHIPs participate in the trial (Baden-Württemberg, Bavaria, Bremen, Hesse, Lower Saxony, North Rhine, Saarland, Schleswig-Holstein, Westphalia-Lippe). Based on the procedure described above, eligible GPs will be contacted by their respective regional ASHIP with a letter containing social norm feedback and in case of intervention groups B and C an invitation to participate in an online training or an online peer-moderated training.

### Randomization

Randomization into four groups (control group and three intervention arms A-C) will be stratified by regional ASHIP and will be carried out by the Zi that is not involved in study conduct and implementation. For randomization, the sql-code, which assigns a random number to all included GPs, is used. The individual GP-random number combinations, ranging from *n*_*i*_ to *m* (number of all GPs that need to be included), are sorted according to the following scheme: If the random number is between 1 and *n*_1_, the corresponding GP is assigned to Group A (*n*_1_= defined number of GPs in that group), if it is between *n*_1_+1 and *n*_2_, the GP is assigned to Group B. The procedure for group C and the control group is analogous, whereas *m*-*n*_i_ describes the size of the control group. GP-random number combinations greater than *m* are not assigned to any group.

### Blinding

Participating GPs will be blinded in the sense that they are not informed about the study and the different interventions. Due to the use of routine health data, patients will not be aware of study participation and data collection. Statisticians will be blinded, but not study staff.

### Control group

GPs randomized to the control group will not be contacted or informed about the study. Hence, they will treat their patients as usual.

### Interventions

We test three interventions against the control group: (A) personalized feedback on own prescribing behavior in comparison to GPs of the region within a visual attention-grabbing graph (social norm feedback), (B) feedback like (A) plus an invitation to participate in the project’s own online training (eLearning) on rational prescribing practice and communication strategies, and (C) feedback like (A) plus an invitation to participate in an online peer-moderated training in quality circle format on the topic of rational antibiotic prescribing, communication strategies, and development of individual starting points for a sustainable behavior change. In detail, the interventions are designed as follows:

- *Group A* receives personalized, individual prescription feedback, which compares the GP’s prescription behavior relative to colleagues in the region. This is complemented by a simple but appealing graph of one’s own prescription quantity compared to the average prescription rate of peers in their region (social norm feedback). This shall encourage to critically question one’s own prescribing behavior.
- *Group B* receives personalized, individual prescription feedback as in (A), and in addition, an invitation to participate in a project-specific online training. The online training fulfills two requirements that initially seem contradictory. It must be generalizable and thus ensure broad application. At the same time, it must address the various causes of inadequate prescription practice. It is also known that for the group of high prescribers a “one fits all” approach cannot be effective since according to Rodgers they belong predominantly to the group of “late adopters” [14]. This apparent paradox for the design of the online training is resolved by a modularized intervention concept. The aim is to provide an individualized portfolio of relevant training modules for each participant in an automated way, which addresses, among other things, the main phenomena of non-rational prescription of antibiotics described in the literature. These include (a) dealing with unavoidable diagnostic uncertainty [15], (b) the mostly unfounded fear of making mistakes, (c) perceived prescribing pressure from patients [16, 17], (d) basic clinical misconceptions [18], (e) the use of treatment algorithms inappropriate to the setting (e.g., treatment regimens of hospital care applied to primary care), and (f) rational consideration of practice characteristics, such as a high proportion of particularly vulnerable patient groups (e.g., due to demographic characteristics, a high proportion of inpatient care facilities, or deprived population groups) [15]. Participating GPs undergo an initial assessment at the beginning of the online training. This assessment captures GPs’ attitudes and individual awareness of the problem of inappropriate antibiotic prescriptions. A pre-developed, weighted algorithm considers the respective information provided by the training participants and proposes an individually tailored module portfolio. For optimally addressing participants, the Transtheoretical Model of Behaviour Change (TTM) by Prochaska and Di Clemente is taken into account [19]. The TTM describes several stages on the way to a change in behavior. It originates from behavioral psychology and is used in interventions to change health behaviors, e.g., tobacco smoking and alcohol consumption, but also in changing medical (prescribing) behavior [20, 21]. The content of the modules focuses on knowledge transfer (e.g., guideline update, antibiotic substances), problem-solving strategies (e.g., doctor-patient communication, intercultural communication, safety netting), everyday strategies (e.g., medical documentation, integration of point-of-care tests, and delayed prescribing) and content that encourages reflection (e.g., evidence-based medicine, medical ethics). Depending on personal interest and recommended modules, the duration of the online training is estimated to range between 90 and 180 min (45 min minimum). If interested, participants can ask for information material to be sent to them afterwards.
- *Group C* receives personalized, individual prescription social norm feedback like (A), and in addition, the invitation to participate in a peer-moderated online training similar to quality circles. These peer-moderated trainings are each moderated by a GP colleague (peer), who already has experience in moderating quality circles. To prepare for the peer intervention, the peers undergo a specific online training. In the first part of the training, peers participate in online training on the intervention (B). In the second part, the peers are trained in three areas according to current medical-didactic findings [22, 23]: giving constructive feedback, motivational interviewing [24], and dealing with group dynamics in heterogeneous groups. The latter is also relevant regarding “difficult participants” in order to recognize and address resistance within the group. The trained peers then moderate the exchange with the participants in an online training and provide content of the individual modules according to their needs. Here, too, change management is to be initiated using TTM. For this purpose, the peer discusses value concepts as well as the individual problem awareness for inadequate antibiotic prescriptions within the group at the beginning of the event. The peer can thus focus and reflect on the intervention content according to the group specifics, both on the cognitive level and on the behavioral level.

Both, the online training and the peer-moderated online training will be CME (continuing medical education) accredited. Also, participants will receive an expense allowance. Overall, the intervention targets the entire group of patients treated by participating GPs for an illness that may involve the administration of an antibiotic.

### Harms

Harms are not expected as the careful prescription of antibiotics is based on national clinical guidelines.

### Outcomes

**Fig. 1:**
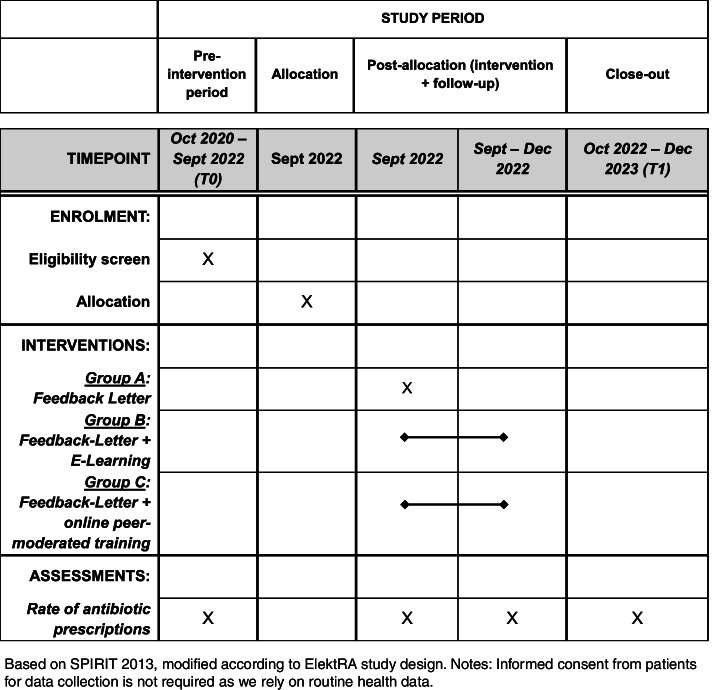
Schedule of enrolment, interventions, and assessments

The primary outcome is the overall rate of antibiotic prescriptions, measured as the proportion of patients with antibiotic prescriptions out of all patients with prescriptions within the nine regional ASHIPs before intervention (October 2020–September 2022) and after it officially starts (October 2022–December 2023). All interventions are completed by December 2022. The changes in prescription rates between those two-time frames are compared between the control group and the interventions. Effects measured at this level are very robust, as neither imprecision in diagnosis coding (ICD 10) nor possible changes in coding behavior have an influence on this outcome. The same applies to the secondary outcomes. Secondary outcomes are the overall prescription rates before and after the intervention for cephalosporins and fluoroquinolones, respectively. The choice of these drug groups is based on the European Surveillance of Antimicrobial Consumption (ESAC) quality indicators for the outpatient sector [25] See Fig. 1.

### Sample size

**Fig. 2:**
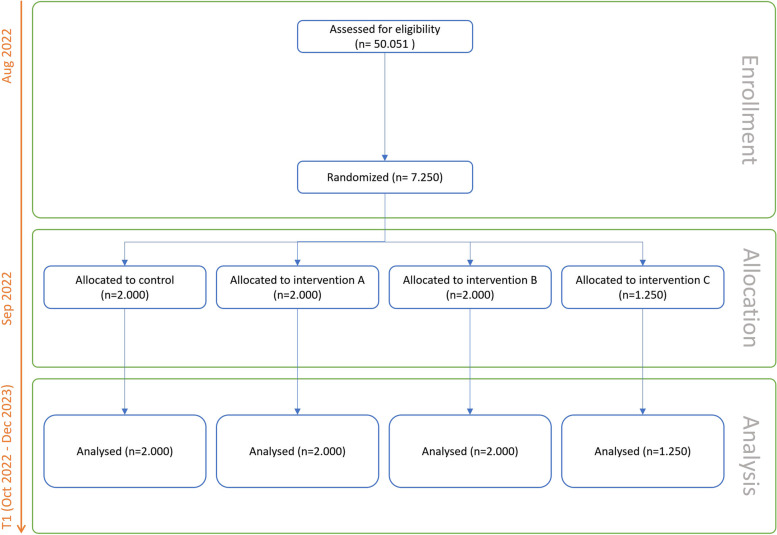
CONSORT flow diagram, estimations on eligibility are based on data of 2020, T0 data from October 2020 to September 2022 is analyzed retrospectively. Source: http://www.consort-statement.org/, Version of 2010 (12.11.2021)

We assume that a total antibiotic prescription rate of 12% (rate of high prescribers in the predecessor project RESIST, unpublished data) will be observed in the control group. This rate is expected to be reduced to 8.7% in group A, to 8% in group B, and to 7% in group C. Since all interventions shall be compared to the control, we adjust for multiple testing via the Bonferroni correction and hence the type 1 error is set to 1.67% (two-sided). A Poisson model was used for sample size calculation. To be able to show the effect assumed for the comparison between the control group and group A with a power of 80%, 2000 practices are required in both groups. Due to the feasibility, maximal 1250 practices can be included in group C for comparison to the control. Thereby a power of 98.8% can be gained. Group B consists of 2000 practices to include all remaining eligible practices in all nine participating regional ASHIP. For the comparison between the control group and group B, a power of 94.6% can be gained. This sample size calculation refers to the intention-to-treat (ITT) population. This population includes all randomized practices regardless of whether they accept the offer of training in groups B and C. However, we are also interested in the per-protocol (PP) population which is analyzed within a secondary analysis. The PP population includes only those practices in groups B and C that participated in the training courses according to protocol. Thus, only the assumed effects between the control group and groups B and C change. We assume that in group B, the rate in this population can be reduced to 7.5%. Furthermore, we assume a participation rate between 30% and 40%, i.e., between 580 and 800 practices participate in the online training. Assuming this difference and the Bonferroni adjusted type 1 error of 1.67 a power between 80% and 89% can be gained. In group C, we assume a participation rate between 17% and 20%, i.e., between 215 and 250 physicians will presumably participate in the peer-moderated online training. Under the assumption of a reduction to 6% and the Bonferroni adjusted type 1 error of 1.67 a power between 80% and 85.8% can be gained See Fig. 2.

### Data collection, completeness, and quality

The data used for the study include all antibiotic prescriptions for patients with statutory health insurance (SHI) that were filed at a pharmacy. In addition, the outpatient claims data of the participating SHI funds (according to §295 SGB V) are used for the diagnosis information of the included patients. Thus, the trial is based on pseudonymized routine data based on GP level. Information collected includes age groups and sex of patients, ARTI diagnoses (grouped and individual diagnoses), antibiotic prescriptions, group affiliation of the physician (intervention vs control), region, and level of urbanization. Thus, a fairly complete data set is available on each GP and the patients treated in the respective practice. However, since the data are not collected for scientific reasons, there are some relevant limitations: The information on the diagnosis depicts the coded morbidity according to ICD10-GM. In particular, the severity of the disease or the patient’s condition may not be adequately reflected by the coded diagnosis. Moreover, there is no connection between diagnosis and prescription in our data. Therefore, we have developed an algorithm that allows a reliable association between diagnosis and prescription. First, different inclusion and exclusion diseases are defined based on ICD-10 diagnosis codes. A patient may not have received both an inclusion and an exclusion diagnosis within a quarter. Furthermore, only one disease group (e.g., upper respiratory tract infections) may have been coded within a quarter. In order to exclude misclassifications of initial prescriptions due to severe courses, patients diagnosed by physicians of different specialties are excluded. To ensure the most accurate assignment of diagnoses and prescriptions, the diagnosing practice must also be the prescribing practice. This rule is only deviated if the pseudonym of the prescribing practice has not billed any case in the entire calendar year. In this case, an error in the pseudonymization is to be assumed and the prescription is automatically attributed to the diagnosing practice. However, we still do not know whether the prescription was only filed or actually taken by the patient. This could lead to a slight overestimation of the actual antibiotic consumption among patients in outpatient care.

### Statistical methods

All prescription rates for the primary and secondary outcomes are presented descriptively for the entire sample and separately according to groups. Sampling units are practices. A negative binomial regression model will be used to evaluate whether the change of prescription rates differs between control and intervention(s) (primary outcome). The negative binomial model is used because we assume that the mean and variance for prescription rates will be different (also seen in the RESIST study). Hereby, the number of antibiotic prescriptions after the intervention(s) is considered as the dependent variable, the number of patients is treated as the offset variable, the grouping variable and the area of the respective regional ASHIP are fixed effects, and the rate before the introduction of the intervention(s) is a covariate in the model. Although we used the Bonferroni-corrected significance level within the sample size calculation, we will evaluate the primary outcome with the less conservative Dunnett test which uses the information that the three interventions will be compared to the same control. The resulting adjusted *p* values will be compared to the global significance level of 5%.

The secondary outcomes will be exploratively analyzed using a negative binomial model without adjustment for multiple testing. Thereby, the dependent variables are the prescription rates for cephalosporins and fluoroquinolones, respectively. The fixed effects are the grouping variable, the area of the respective regional ASHIP, and the rate before the introduction of the intervention(s) for cephalosporins and fluoroquinolones, respectively. The number of patients serves as the offset variable.

The ITT population will be primarily used for all analyses. Thereby, we can answer the question about the effect of offering online training or peer-moderated online training. In a further explorative analysis, the comparisons are repeated in the PP population to examine the effects according to the training received. This population includes all practices in which the GPs participated in the training offered. Only the result of the primary analysis in the ITT population will be interpreted in a confirmatory sense. The results will be reported according to the CONSORT statement [26]. Incidence rates, rate ratios, associated 95% confidence intervals, and *p* values will be reported. The analysis is done with standard statistical packages such as SAS, Stata, R, or SPSS [27–30]. In addition, explorative subgroup analyses will be conducted in both the ITT population and the PP population, relating to the effect of the intervention on prescription rates or drug selection for individual indications (respiratory tract infections, urinary tract infections). In an extended multivariable analysis, reasons for an indicated prescription are also taken into account as well as patients’ multimorbidity, failure of previous therapy, or the size of the practice. If the number of cases is large enough, regionalized analyses will also be performed.

### Risk of bias

There are several risk factors that could influence the success of the project: It is possible that there is too little acceptance on the part of the GPs contacted, resulting in low-participation rates in the interventions offered. In the case of peer-moderated training, this could lead to heterogeneous group sizes and the desired intercollegiate exchange not being able to take place due to a low number of participants. To counteract this, a sufficiently high number of GPs is contacted, and the content of the letters is closely coordinated with the regional ASHIP experienced in this field. Participation is also incentivized by an expense allowance. In addition, the training courses are conducted online in a format similar to quality circles to maximize acceptance among GPs. Another external risk factor that cannot be influenced is a higher or lower level of disease activity in the period under review (wave of flu, Sars-CoV-2 pandemic, other disruptive effects, or overlapping events) than in the preintervention period used for the evaluation. As a countermeasure, in addition to a pre-post comparison with an extended comparison period, a comparison to non-contacted high prescribers in the participating regions (Group D as a “real control group”) is also carried out here.

### Process evaluation

The process evaluation of the interventions tested in ElektRA is carried out using a mixed-methods approach, i.e., combining qualitative and quantitative research methods. The focus is on the question of how the intervention contents are perceived, accepted, and implemented in everyday practice by the GPs. In addition, the non-participants, i.e., non-responders to the project or refusers of the interventions, will be examined more closely. The question, which barriers prevail both in terms of participation and reducing the prescription of antibiotics where it is appropriate and safe will be investigated.

The selection of the participating GPs for the qualitative survey follows the principles of purposeful sampling, i.e., the composition of the sample corresponds to certain socio-demographic criteria (e.g., age, gender, practice size, degree of urbanization). At the heart of qualitative research is a systematic, open approach both in terms of study design and data analysis. To analyze GP readiness for implementation, 1–3 (telephone) interviews per participating ASHIP and per intervention group will be conducted with the participating physicians. With 9 participating regional ASHIP, about 70 interviews will take place. According to our experience, these will have a duration of approx. 20–30 min. The interviews will be conducted by an experienced interviewer. All interviews will be audio-recorded, pseudonymized, and fully transcribed. The data collection will be finished as soon as the generated hypotheses are saturated [31]. The analysis takes place at the content level. In the end, a system of categories is created that allows for summarizing the most important results of the interviews. The analysis is software-supported (e.g., with NVivo 10).

The results of the qualitative study will be transferred into a questionnaire in the following, which will be adapted for the three intervention groups. In this way, the hypotheses developed in the qualitative process evaluation will be investigated on a representative sample of the participating GPs. Further, to reach the non-responders, a questionnaire (as low-threshold as possible) will be sent to all high prescribers (within groups B and C) who have decided not to participate in the respective training offered. In this questionnaire, participation in an in-depth interview is advertised.

### Study registration

The study has been registered with Current Controlled Trials Ltd. with the reference ISRCTN95468513.

### Trial status

In August 2022, final work on the implementation of the online training will be completed and all project documents will be prepared. Recruitment of participants will start in September 2022.

## Discussion

The focus of the study is the implementation of individualized, low-threshold interventions in an elementary area of drug therapy in standard care. The non-indicated use of antibiotics or the use of inadequate substance classes has direct consequences for the individual treated as well as medium- and long-term consequences for society in general. In addition to the indirect threat of bacterial resistance, non-indicated prescriptions of antibiotics are also associated with immediate risks for patients: Adverse drug reactions (ADRs) occur in up to a quarter of all antibiotic prescriptions and antibiotics account for 20% of all emergency admissions for ADRs [32].

ElektRA is based on the results of several international studies on the reduction of inappropriate antibiotic prescriptions in primary care [7–10, 33–35]. Using different kinds of social norm feedback, these studies were able to demonstrate significant reductions in antibiotic prescriptions, in large part to a clinically relevant extent [7–10, 33]. However, there are no studies to date on whether and with which measure it is possible to get physicians with immoderate prescribing behavior in the primary care sector in Germany to prescribe less or in a more targeted manner. Existing studies mainly come from the UK [7, 8, 35], USA [10], and Australia [9], none of which is comparable with the German health care system. In the UK, for example, most physicians are directly employed by the National Health Service, which makes it easier to enforce targets regarding prescription rates. Also, especially the group of high prescribers tends not to participate in existing projects and efforts targeting the improvement of prescribing behavior, presumably because there is no or only a low awareness of the individual problem, or no general interest in the topic. If interventions investigated within ElektRA show effects, the knowledge gained can be transferred to standard care with only minor restrictions and can also be used for other problematic prescription fields. Within the German health care system, ElektRA can thus be regarded as a pilot for a targeted, science-based prescription management.

Apart from the fact that, to our knowledge, there is no study in Germany that explicitly focuses on high prescribers, we are also not aware of any study that investigates the influence of social norm feedback (with and without further intervention offers) on prescribing behavior. ElektRA allows statements to be made about whether GPs with inappropriate prescribing behavior are more likely to be reached with an intervention that can be completed anonymously and flexibly in terms of time and that allows them to focus on their own topics (intervention B) OR with an intervention in which they can exchange and discuss their own problem areas among peers (intervention C). In addition, ElektRA may provide information on whether these two interventions have more influence on prescribing behavior than simply mirroring one’s own prescribing behavior in a comparison to peers (Intervention A). Last, but not least, ElektRA is designed to provide insights into how well-tailored training measures are accepted by those they have been especially designed for.

From a methodological perspective, using a real control group is a major strength of ElektRA. This is rarely the case in randomized controlled studies in health care research. Since the project is not made public and no informed consent must be obtained by GPs in advance, neither the participants nor the control group knows that their prescriptions are monitored as part of a research project, which massively reduces the risk of distorting effects and excludes selection bias, for example.

Using individual feedback is likely to promote awareness of the problem of inappropriate antibiotic prescribing and may form the basis for behavioral change. At the same time, there is also the danger of defensive reactions to reduce the cognitive dissonance that has arisen. To mitigate defensive reactions as far as possible, the social norm feedback is tested in a pre-test before the interventions start. In addition, the reasons for defensive reactions are to be determined within the framework of the qualitative evaluation, thus generating important findings for future implementation in outpatient care. Changing clinician decision-making among otherwise difficult-to-reach high prescribers leads directly to an improvement in care, as inappropriate antibiotic prescribing is reduced [5]. The increase in quality is achieved both in terms of the quantity of antibiotics used, as well as the substances used. The quality of treatment for the individual patient is promoted. At the same time, the study aims to ensure the effectiveness of antibiotics by preventing further development of antimicrobial resistance.

## Conclusion

The rational use of antibiotics remains a highly relevant topic for actual and future health care. This is also shown by the large number of projects that have already been (successfully) implemented in this area. However, these projects are usually based on voluntary participation, resulting in an overrepresentation of physicians with an already moderate antibiotic prescribing behavior and failing to reach GPs with antibiotic overuse. For this reason, ElektRA specifically addresses high prescribers in order to nudge a sustainable change in their behavior. For the first time in Germany, the project is testing and comparing systematically whether and which approach/training format can succeed in gaining access to the group of high prescribers and which effects can be achieved in routine health care. It can also provide insights into the reasons why the group of high prescribers may not be reached. If effective strategies for high prescribers are established within the project, they can be applied not only to the antibiotics addressed here, but also to other areas of prescription management.

## Supplementary Information

**Additional file 1.**

## Data Availability

Not applicable, as no data has been collected yet. Inquiries about the study intervention can be made to Prof Attila Altiner (attila.altiner@med.uni-heidelberg.de). Results of the study are published at scientific congresses and in scientific journals as well as in relevant newspapers with science sections.

## Abbreviations

ADRs: Adverse drug reactions
ARTI: Acute respiratory tract infections
ASHIP: Associations of Statutory Health Insurance Physicians
CME: Continued medical education
ESAC: European Surveillance of Antimicrobial Consumption
GPs: General practitioners
ICD 10: International Statistical Classification of Diseases and Related Health Problems
ITT: Intention-to-treat
PP: Per-protocol
TTM: Transtheoretical Model of Behaviour Change
Zi: Central Research Institute of Ambulatory Health Care in Germany
